# Treatment of Dental Caries, Complicated with Disorders of the Pulp and Peridental Membrane

**Published:** 1852-10

**Authors:** Robert Arthur


AMERICAN JOURNAL

OF

DENTAL SCIENCE.

Vol. III. NEW SERIES—OCTOBER, 1852. NO. 1.

### ARTICLE I.

*Treatment of Dental Caries, complicated with Disorders of the Pulp and Peridental Membrane.* By ROBERT ARTHUR, M. D., D. D. S.

No. VI.

WE come now to the consideration of the last step in the operation which has so long engaged our attention. I have given an account in as full detail as I thought consistent with justice to the subject, without being too diffuse, what I conceived to be the best means of accomplishing the entire removal of the pulp. It now remains to describe that part of the operation deemed essential to its completion, viz. filling the fang or fangs. This is a comparatively simple part of the whole process, but there are points about it to which some attention may be given with advantage to those who have not had experience in the practice of this operation.

The instruments for filling the fangs, though exceedingly simple in form, require to be made with care. Piano wire is more suitable for this purpose than steel of any other kind or in any other form of which I am aware, and steel is decidedly preferable to any other metal. These instruments do not differ in form from those already described, for the removal of the pulp, except that no barbs are cut upon them, but on the contrary they are made perfectly smooth by burnishing and giving them a spring temper. Except when the canal of the fang, to be filled, is flattened, they should be made round so as to obtain the greatest strength with the least bulk. A number of them should be prepared, and graduated from the smallest diameter of the fang to be filled to the largest size required by the canal at its largest diameter. Tempering such small instruments is, I am aware, a nice operation, but it renders them so much more available for this purpose, that no pains ought to be spared to accomplish the object.

The best material for filling the fangs is, undoubtedly, gold; it is better suited, in every respect, for this purpose than any other material with which I am acquainted. I see, however, no objection to the use of tin foil for this purpose—it cannot be consolidated so perfectly as gold, but it can be made sufficiently compact to answer the purpose. But gold, as I have just said, is preferable to any other material for the purpose; it can be used more conveniently and can be rendered more compact with slighter compression.

The facility with which gold may be employed for filling the fangs depends upon the form and manner in which it is used. No. 30, is undoubtedly best adapted for this purpose. Whatever may be thought of this gold, (which I introduced to the profession, and which I have used for nearly two years, and which I still use, almost exclusively, with increasing satisfaction,) for ordinary operations, for filling the fangs, it will be acknowledged upon trial, I am sure, invaluable. For this purpose, indeed, Dr. Maynard, to whom I have had occasion so often to allude in these papers, has used gold of this kind for a number of years.

By Dr. Maynard it is cut into a narrow strip, one end of which is carried to the extremity of the canal of the fang with a suitable instrument, which is withdrawn, leaving the gold in place; a fold of the gold near the extremity is taken by the instruments, are carried up to the end, and so, fold after fold is condensed against the first until the fang is filled.

I will describe somewhat minutely my own method of procedure, which I find after trial of other suggested methods, enables me more conveniently and perfectly to accomplish the desired object.

I first cut the gold from a narrow strip into small oblong squares, about two lines in length, and one in width, which I lay in a convenient place for use. I then set in order the instruments I intend to use for the purpose, and take care to provide several of the smallest size. I then wash out the pulp cavity and fangs with the sponge, and pass the smaller instruments into the fangs to be filled to be sure that they pass readily to the point to which I wish the gold to be carried. The cavity and canal of the fang are then to be carefully dried, which may be done by twisting a small piece of cotton around one of the barbed instruments used for removing the pulp, and passing it repeatedly into the fang. It will be necessary to change the cotton several times, or have several instruments prepared in the same way, as the small quantity which can be used soon becomes saturated.

There are two methods of placing these small pieces of gold in a position where they can be carried conveniently into the fangs—I hope these minute details will not be considered trifling and unnecessary, to many I am sure they will not be so thought, if I judge from my own experience—one is to take up the gold with a pair of spring forceps and place it in the cavity of decay near the opening of the fang, from which place it is carried into and up the fang with suitable instruments—the other is to place the gold on a piece of wood and take it up by sticking the instrument through it, which after some practice will be found a very convenient way of getting over a vexatious little difficulty.

The canal of every fang is a lengthened cone—in some parts it is a simple tube, but from the opening from the pulp cavity to the foramen at the extremity of the fang it is always more or less conical. In filling the fang it is always advisable, in using the small pieces of gold which I have recommended, to carry them into the canal of the fang with an instrument but slightly smaller than the opening from the pulp cavity. As soon as it has passed the opening of the canal as far as the instruments used for introducing it into the canal will go, a smaller instrument is taken with which the gold is forced up somewhat farther until it is again stopped by the narrowing diameter of the canal, and so on till the instrument which was previously ascertained to be sufficently small to pass to the extreme end of the fang, can be used and the piece of foil carried up to its place. After this piece is as well condensed as it can be, another similar piece is to be taken, introduced in the same manner and gradually carried up to its place. The length of the pieces of gold foil may be increased with every piece used, and when the diameter of the canal is large enough, as it is filled toward the opening to permit the use of strong instruments and considerable force, strips of the same kind of gold may be employed with advantage.

I make use of gold foil in the manner described in the more contracted parts of the fangs, for this reason: the canal is liable to become choked with the gold, if too much is used at once before it reaches the desired point, and when this occurs, it is impossible, with the necessarily delicate instruments employed, to force it further up or to remove it. The advantages of first using stouter instruments than will reach the extreme point of the fang, and as the gold is carried up taking the finer and finest instruments, a little reflection must make obvious. The smaller instrumennts would pass entirely through the piece of gold in every attempt to force it into the fang at the place where it is of much larger diameter than the instruments, but as the canal of the fang contracts, the gold is drawn together so as to present a body offering greater resistance, and the finer instruments will then have the same relation to the diameter of the canal, as the larger one when it was larger.

This is my general plan of procedure. It is apparent, that success in this part of this delicate operation requires time, perseverance and careful, patient manipulation. All this, however, is required, daily, in the ordinary faithful practice of our profession, and the man who is afraid to encounter obstacles, and patiently labor to overcome them, will never reach any desirable eminence in this or any other profession in which he may engage. If, however, he is willing to devote himself, in the right spirit, to such labor as this in which I am endeavoring to assist him with my own experience, he will obtain results which will fully reward him for his pains.

The fangs most easily filled are those of the superior incisor and canine teeth. If the directions above given are put into practice, the most inexperienced operator will be enabled to fill these teeth very perfectly. The palatine fang of the superior molars are generally cylindrical, and when the external opening is favorable, is not difficult to fill. This is also the case with the posterior fang of the lower molars. Since the publication of the last number of these papers, my attention has been called to an error which it is proper in this place to correct. I stated that generally the fangs of the lower molar teeth were flattened, so as to form in each two canals, one presenting toward the tongue, and one toward the cheek. This is the case with the anterior fang; but generally the posterior fang has but one canal in the centre, sometimes cylindrical, sometimes more or less flattened, and sometimes formed like the canals of the anterior fang.

After filling the fang or fangs of any particular tooth under treatment, I always consider it advisable to allow a few days to elapse before filling the pulp cavity and cavity of decay. The irritation caused by the force necessary to be exercised for this purpose is always liable to produce a slight degree of inflammation of the peridental membrane, as it is at this time predisposed to inflammation, and time should be allowed for any inflammatory action to subside. If this inflammatory action should exhibit itself in tenderness to pressure upon the tooth, it will always be advisable to wait till this passes away, and when necessary, by the use of counter irritation along the course of the canal to assist in its dissipation.

Up to the present moment, we have been engaged in this consideration of the treatment of teeth in which the pulp still retained its vitality, and which, as a part of the process it was necessary to remove. In the treatment of such cases, I have met with so much success in my practice by pursuing the method which I have described, that I never anticipate trouble or inconvenience as a consequence. Slight inflammation of the peridental membrane, being all the trouble which is likely to occur; and this, with the least attention, can in every case be subdued with little trouble. In most cases no treatment at all is necessary.

But another class of cases frequently come into our hands which require particular notice; those in which the pulp is already deprived of vitality, or entirely decomposed. With teeth in this condition, which at first sight would seem to be easy to treat, trouble frequently follows the operation of filling. Why this should occur more frequently when the pulp is dead than when it is obliged to be destroyed whilst in full vitality, is a question well worthy of attention.

Teeth in which the pulps are dead may be divided into five classes:

I. Those which are perfectly healthy.

II. Those which are in such condition that even slight causes of irritation will give rise to peridental inflammation.

III. Those of which the peridental membrane is already in an inflamed condition.

IV. Those which are the cause of alveolar abscess discharging externally.

V. Those in which the matter formed is discharged through the canal of the fang.

I. Teeth will not unfrequently be met with, of which the pulps are devoid of vitality, and in some cases entirely wasted away, and such teeth may be found in perfectly healthy condition. They may be considered in four classes:

1st. Such as are sound or only slightly decayed.

2nd. Such as may have been filled when but slightly carious, or, at least, before the caries has reached the pulp.

3d. Such as may have been filled upon the exposed pulp.

4th. Such as have decayed to the pulp, which has been destroyed by the influence of foreign substances.

1st. The vitality of the pulp is frequently destroyed by a blow upon a tooth, which may either break off its connection with the jaw, or so injure the chord passing from the fang as to cause a high grade of inflammation, which, passing down to the pulp may occasion its death and disorganization. Very commonly a very high degree of peridental inflammation also occurs proceeding to alveolar abscess, but I have a number of times met with teeth deprived of vitality by this cause, which were in healthy condition; as far, at least, as their connection with the general system was concerned. In some of these cases no trace of previously existing alveolar abscess could be described, nor could it be discovered from the account of the patient that so high a degree of inflammation at any time existed as to have occasioned severe pain. But, even where alveolar abscess has occurred, the parts in time frequently perfectly recover their healthy condition. Teeth in this condition, after the lapse of years, invariably, so far as my observation goes, become more or less discolored, and very frequently the only evidence, besides this discoloration, that there is anything wrong about them, is the wasting away of the gums and edges of the alveolar process. Wherever they occasion any disfiguration they should be treated by making an opening at a convenient point into the pulp cavity, and removing the remains of the pulp, and as much of the discolored bone toward the anterior surface, as can be taken away without risk of making the tooth too frail to bear filling. As it is the dentine and not the enamel which is discolored, the gold underneath will give it a slightly yellowish, but much lighter hue. I have treated such cases in this way with great satisfaction, and have frequently left them with a difference of shade so slight as scarcely to be perceptible to an ordinary observer. It has been proposed in such cases, (by Dr. J. H. Foster, I believe,) to place a piece of white paper just underneath the enamel and to fill up against it; this is certainly an elegant operation, and I see no reason, if care is taken to keep the paper perfectly dry, why it should not succeed.

But teeth will sometimes be found dead, as they are called, and the loss of vitality of the pulp cannot be accounted for in the way above mentioned. I mean, of course, before the pulp has been reached by decay. They may, in this case, be either slightly decayed or perfectly sound. It is an undoubted fact, that the dental pulp is sometimes, though rarely, deprived of vitality by causes of which it is difficult to give a satisfactory account. Further data are necessary to clear views upon this point. Whether there is a spontaneous absorption of the substance of the pulp without sufficient inflammation to occasion pain, which can be recollected by the patient, is a question which I cannot now determine. It is certain that no trace of pulp, except a small quantity of dry substance, can, in some of these cases, be discovered either in the pulp cavity or canal of the fang, and on inquiring of the patient it will be found in some cases that no painful sensations of any kind have been experienced about such teeth. Whether this information is to be relied upon I cannot now determine. It is certainly possible that in some disordered inflammatory condition of the general system, the vitality of a certain tooth predisposed to inflammation, may be destroyed without attracting the attention of the patient at the time, but it is questionable whether in a state of ordinary health this is likely to occur. In many cases, although no severe pain has occurred, I have reason to believe that some uneasy sensations are experienced from time to time, occasionally amounting to pain.* I have never known any difficulty to follow the operation of filling the fangs of such teeth.

2nd. Such teeth as may have been filled when but slightly carious, or at least before the caries has reached the pulp.

The death of the pulp, in such cases, may be attributed to several causes: to pressure upon a thin plate of bone separating the pulp cavity from the cavity of decay; to impressions conveyed to the pulp already in an irritable condition, through the

*In a late conversation with Prof. Harris, I found that his attention was drawn to this fact some years ago; that he has made careful observations with reference to it, during his long practice, and will give the results of his experience in a forthcoming edition of his "Principles and Practice of Dental Surgery," which is now in press. Dr. Maynard, too, mentions some interesting cases of this kind which have passed through his hands.

medium of a metallic filling, by substances much below or above temperature of the blood taken into the mouth; or to hard pressure upon highly inflamed dentine in the vicinity of the pulp, as shown in case III. Generally in these cases severe pain follows the operation of filling, and the inflammation of the pulp occurring, terminates in alveolar abscess. Sometimes, however, inflammation terminates in resolution, and after a time the parts recover their healthy tone. Sometimes after a lapse of time an alveolar abscess will heal spontaneously and the parts will be found in healthy condition. In these cases the indications are the same as in those just described. The probabilities of a successful result are the same.

3d. Such teeth as may have been filled upon the exposed pulp.

It is to be hoped that such cases as these will not be so frequently encountered in the future as in the past. Practitioners of a different stamp from those which have heretofore infested it in great numbers, are coming into the profession now; they will be too well instructed to do anything like this ignorantly, and, I trust, if deterred by no higher motive, will see too clearly the policy of honest practice to do such a thing with their eyes open. If the pulp is exposed at the slightest point, and the cavity of decay so filled as to allow the filling to come in contact with it, the death of the pulp will be an inevitable consequence. But, like the class above indicated, although the peridental membrane will not escape inflammation, it may, and sometimes does, recover healthy action, and the teeth may be treated with good hope of success.

4. When the pulp becomes exposed by the action of ordinary decay to contact with foreign agents, and the pulp cavity is allowed to remain open, there is more apt to be continued irritation and inflammation. This may continue to be the case after the pulp is entirely dead—it will almost certainly be so if the opening of the canal at the alveolar extremity of the fang is not very small. This will be occasioned by particles of food or other substances being forced through the fang so as to come in contact with the peridental membrane. But, in many cases, such teeth will be found in healthy condition, and with ordinary precaution may be filled, with every prospect of their preservation.

II. Notwithstanding the division I have made of these cases, all the teeth in the condition just described must be regarded as liable to inflammation from slight causes; the force used in filling, as I have already intimated, will be often sufficient to produce inflammation of a high grade. But such as already give evidence of peridental inflammation, however slight, as will be evinced by their looseness and tenderness to pressure, are particularly liable to subsequent severe inflammation.

This sub-inflammatory condition, if it may be so designated, is not always an indication, however, that this operation will be followed by unpleasant consequences, for the irritation in many cases, is kept up by the presence of putrid and acrid matter in the pulp cavity and fangs, occasioned either by the decomposition of the pulp, or after this has taken place, by the discharge of fluid of some kind through the alveolar foramen of the fang into the internal cavity, or the decomposition of particles of food. Hence, it will often be found that this operation instead of increasing the inflammatory action will completely cure it. A common cause of irritation, as already intimated, is the forcing of food and other foreign substances into and through the fang if the cavity of decay remain open, and the removal of the cause of irritation in such case will almost invariably effect a cure. Of this fact the following case is a fine illustration:

Mr. B., æt. 47—May, 1851.—The two central incisors had been dead, as it was said by the patient, for more than thirty years, the cavities of decay, now quite large, had been filled, but the fillings had come out. It was not thought advisable by his dentist to fill them again, but as he was exceedingly reluctant to lose them, as they were strong, useful teeth, he determined to make another effort to save them. On consulting me, I agreed to attempt to operate for him in the case. There was at the time considerable inflammation present, and the gentleman informed me that they were somewhat annoying to him a great part of the time on account of their tenderness to pressure. From this cause it was not agreeable to use them in eating, and he involuntary avoided using them. I very carefully, by repeated use of the syringe, washed out the fangs and filled them in the usual manner. There was no increase of inflammation after the operation—for the probable occurrence of which I was careful to prepare him—on the contrary all the inflammatory symptoms passed away, and on seeing him after the lapse of a year he informed me that in biting hard substances he was unable to perceive any difference between these and any of the rest of his teeth. For the wife of the same gentleman I filled two bicuspids, in very nearly the same condition, with the same result. She assured me that until the teeth had been filled they had been uncomfortable to her for a long time.

In these cases where there is every reason to anticipate more or less unpleasant results, it is always best to proceed cautiously, doing as little at a sitting as can be well done, and then to take every precaution against the occurrence of inflammation, where there is evidently very great predisposition to inflammatory action. I generally at the first sitting simply wash out the fangs and remove any extraneous matter which may be in them. I then close up the external cavity with cotton saturated with the solution of sandarach, and direct the patient to call in two or three days. If any inflammation follows this part of the operation, I wait till it subsides, and make use of such means as seem to be indicated to subdue it; when this is accomplished, or, if at the time appointed no inflammation is present, I proceed to fill the fangs, and then dismiss the patient. When all traces of a peridental inflammation disappears, I complete the filling. These may seem to some persons unnecessary precautions, but I am satisfied that many cases have occurred in my own practice in which valuable teeth have been saved by this procedure, or, at least, a great deal of suffering on the part of the patient prevented.

IV. Teeth in which alveolar abscess is present.

It was for sometime a question with me whether such teeth could be treated with a reasonable prospect of success; it is no longer a question, and I do not hesitate to fill them. There are many considerations of importance involved in the pathology of alveolar abscess, which it was my intention to discuss at length in this place, but I must defer it for the present, and simply be satisfied with a few words in relation to the practice to be pursued in such cases.

There is evidently not the same necessity for caution in these cases as those above described; any increase of inflammation here is rarely attended with painful consequences, and it is, of course, always well, when it can be done with safety, to go straight on to the end as speedily as possible.

Very frequently an abscess will spontaneously pass away after the fangs are cleared of irritating matter, and carefully filled. If it should not, it must be lanced deeply, and a piece of cotton or lint placed in the incision, and allowed to remain till it is thrown out by healthy granulations at the bottom, or removed when healthy pus has been discharged freely for a time.

IV. When a discharge of pus or other matter through the canal of the fang is suspected, it will be necessary, in the first place, to ascertain whether it comes from a sac, exterior to the fang, or from some remains of the pulp still in the canal.

It sometimes happens that an abscess will discharge for years through the canal of the fang without causing much, if any, inconvenience to the patient, if the external cavity of decay is open, except when its free egress is obstructed, and then severe pain follows.

In such cases, the attempt ought to be made to put an end to this diseased condition of the parts, and it will generally be successful. Let the fang be filled without any other precaution than to avoid pushing the instruments and gold entirely through the foramen, which, in most of these cases, from the long continued action of corrosive matter, will be found enlarged. Dr. Maynard's very ingenious method of managing such cases I have already described. Whether this is adopted or not, some means must be employed to ascertain, as nearly as possible, the length of the fang, to the end of which the gold should be carried.

When the fang is filled in such a case, the usual outlet of the matter formed is closed up, and it must of course escape somewhere. If let alone it will find its way through the wall of the socket—generally through the exterior wall—but great pain will attend this process. To prevent this an opening must be made near the extremity of the fang, through the external alveolar wall. Before filling the fang, an instrument, giving some indication of its length, should be passed into it and marked: when the operation of filling is completed, this should be used as a means of ascertaining the proper place to perforate the alveolus. This opening for the escape of the pus should be formed at once, as soon as the rest of the operation is completed, for nothing is to be gained by delay; on the contrary, severe inflammation, and great pain will occur. A small sharp drill is sufficient for the purpose. The abscess should then be treated as just described. After all this, it may in some cases be necessary to remove the tooth, and in attempting such an operation the usefulness of the affected tooth is to be considered.

There are cases where the preservation of a single tooth is of so much importance to a patient that it is more than justifiable to attempt an operation which promises very little; there are others upon which it would be useless to waste an ordinary operation. This, of course, is a question to be determined by every operator for himself.

A very curious case occurred in my practice a few years since, which may be mentioned in this connexion. The right superior lateral incisor was filled, but as it was evidently dead and discolored, I advised the removal of the filling for the purpose of enabling me to take away as much of the discolored bone as possible, and also to prevent its further discoloration. On removing the filling I found that the caries had not penetrated to the pulp cavity, which I opened. I then passed a probe to the extremity of the canal; there was no trace of decomposed pulp or fetid matter—no pain was produced. Observed in a moment that the pulp cavity and cavity of decay were filled with what appeared to be saliva. This I carefully dried out, and placed a napkin around the edges of the cavity of decay, over which I supposed the saliva had flowed. In an instant more the cavity was again filled with the same fluid, and I soon discovered that it came from the canal in the fang. I watched it for some time, and after wiping out the fang carefully, I could discover in a very short time the canal and pulp cavity fill with a limpid fluid, which flowed in considerable quantity, coming with distinct impulses, synchronous with the pulsations of the coronary artery of the upper lip, which I could distinctly feel beating under my finger. I filled this fang in the manner just described; an opening already existed through the external wall of the alveolus; when I next saw the patient, which was in the course of a few days, an abscess filled with pus had formed in the place where one had before been. I lanced the abscess, and no further trouble occurred whilst the patient was in my hands. I saw the same patient about a year afterwards, the tooth appeared to be in healthy condition.

Sometimes a portion of the pulp, more or less small, will remain for a long time in the canal secreting pus, which is discharged into the pulp cavity. The presence of such a portion of pulp will generally be detected by the pain occasioned by contact with an instrument passed into the fang, even if the discharge of pus is not sufficient to be apparent upon examination. This is first to be got rid of, and it is sometimes difficult to bring into contact with it the arsenic to destroy its vitality. I generally pass the arsenic up to the point on the end of a piece of thread; if its destruction cannot be accomplished by this means it must be removed with an instrument, regardless of the pain it inflicted. In these cases, however, it is necessary to observe well the precautions recommended in the last number, to avoid passing the instrument both in making the examination and removing any remains of the pulp, entirely through the foramen.

A discharge of matter through the fang, from the cause here mentioned, offers no other obstacle to the operation, than that the parts are in a more or less morbid condition, which must render them liable to inflammation from the irritation attendant upon what is done. Every care must be taken to prevent this or to subdue it when it occurs.

There is still a very important point to be considered before closing, that is, the age at which this operation may be attempted with propriety. It is very obvious that no success, but, on the contrary, bad results must follow this operation if attempted upon teeth before the fangs are fully formed, and unfortunately such cases frequently occur. This question was considered by Dr. J. D. White in a paper published some years ago in a medical journal; I should be glad to see it republished. I cannot at present recollect the paper alluded to particularly, whether it gave any precise data by which to judge of the length of time after the teeth have attained to their full size in the mouth for the complete formation of the fangs. Upon this point, further observation is needed in order to insure the preservation of many teeth of young persons without risk of injurious consequences. Such observations I have not yet been able to make to my satisfaction.

I now feel myself obliged to close these papers, with several subjects scarcely touched, which I had contemplated carefully considering when I engaged in writing them, as indeed is indicated by the title I have given to them. Pressing engagements occupy the whole of my time, and oblige me for the present to set aside this subject. At a future time, not far distant, I hope, if what I have presented should be thought to possess any value, it is my intention carefully to go over the whole ground, and treat, to the best of my ability in all its details, this particular subject, and all which bear any relation to it. I regret that absolute want of time has obliged me to allow a greater part of these papers to go before the profession in a cruder form than I am satisfied to see.

